# SRY gene isolation from teeth for forensic gender identification—An observational study

**DOI:** 10.1371/journal.pone.0294751

**Published:** 2024-01-03

**Authors:** Prathibha Prasad, Mohamed Jaber, Dinesh Y., Prathibha Ramani, Abdulrahman Arafat, Abdalla Khairy

**Affiliations:** 1 Basic Dental Sciences Department, College of Dentistry, Ajman University, Ajman, United Arab Emirates; 2 Center of Medical and Bio-allied Health Sciences Research, Ajman University, Ajman, United Arab Emirates; 3 College of Dentistry, Ajman University, Ajman, United Arab Emirates; 4 Department of Oral Pathology, College of Dentistry, Saveetha University, Thandalam, India; Nair Hospital Dental College, INDIA

## Abstract

Personal identification in forensics is possible with gender determination using DNA (deoxyribonucleic acid) analysis. DNA isolation from teeth samples subjected to extreme temperatures has been shown to predict the gender of the deceased. However, the literature lacks studies on DNA extracted from tooth samples exposed to freezing temperatures. This study aimed to isolate the SRY gene from the extirpated pulp of teeth that were subjected to varying temperatures for gender identification. Thirty teeth with vital pulps, divided into 3 groups were included in the study. Each group consisted of 5 male and 5 female tooth samples. The groups were exposed to diverse environmental factors for three weeks. Group 1: room temperature (R group); Group 2: high temperature (H group) and Group 3: freezing temperature (F group). Later, DNA was isolated from the pulp tissue, and the SRY gene was amplified using PCR (Polymerase Chain Reaction). The Sensitivity and Specificity of the results were analyzed. SRY gene detected in the study samples identified accurate gender with a 46.70% Sensitivity and 93.30% Specificity. Significant difference was found in the correlation between gene expression and gender among the three groups (p = 1.000). The study validates that dental pulp tissue can be a reliable source for DNA extraction. And SRY gene amplification from teeth exposed to diverse environmental conditions. Further investigations are required to validate its application in forensics.

## Introduction

In forensics, gender identification plays a crucial role in personal identification. While morphological traits are effective in identifying genders, their application is compromised in mutilated human remains or when only fragments of the body are recovered [1]. In such situations, Forensic odontologists can help particularly when only a few teeth and skull remain are available. Though various features like crown size, and root lengths can differentiate male and female genders, instances with an absence of dental records and mutilated teeth samples make it impossible to solely rely on the morphological traits [2]. Therefore, in such instances, molecular analysis like DNA fingerprinting or DNA profiling is advantageous [3]. Notably, the extraction of DNA samples for gene expression analysis in gender determination has been studied.

A considerable number of studies have put forth the use of Dental pulp as a reliable source for genomic DNA [4, 5]. This is due to their ability to withstand high temperatures [6]. This protection allows for better preservation of the DNA, even under extreme conditions. Consequently, forensic science utilizes teeth, particularly dental pulp, for gender prediction by gene amplification.

Recent studies on the sex-determining region "Y" gene (SRY), the mammalian Y- chromosomal testis-determining gene that induces male sex determination, have shown promising results in the differentiation of male and female gender [7]. Most researchers have used PCR and real-time PCR for SRY gene amplification. SRY gene was detected from epithelial cell isolates obtained from saliva-stained removable acrylic dentures and shed epithelial cells isolated from toothbrushes [8]. SRY gene was detected from epithelial cell isolates obtained from saliva-stained removable acrylic dentures and shed epithelial cells isolated from toothbrushes [9, 10]. However, in certain cases involving maternal-fetal microchimerism, as well as individuals with a history of blood transfusion or organ transplantation, false-positive results may occur [8].

To date, very few studies have investigated gender determination in teeth exposed to varying temperatures, especially using dental pulp. Therefore, our research aims to fill this gap by detecting the SRY gene in dental pulps subjected to various temperatures, including freezing temperature. Our objectives were to identify the presence of DNA in the pulp tissue, determine the expression of the SRY gene in dental pulp samples stored at varying temperatures such as room temperature (approximately 30°C), high temperature (70°C), and freezing temperature (-80°C), and correlate the SRY gene expression with the actual gender of the subjects.

With this research, we hope this knowledge can enhance the reliability of dental pulp as a source for gender prediction in forensic investigations where varying temperatures may have been encountered.

## Materials and methods

### Sample characteristics

**Fig 1:**
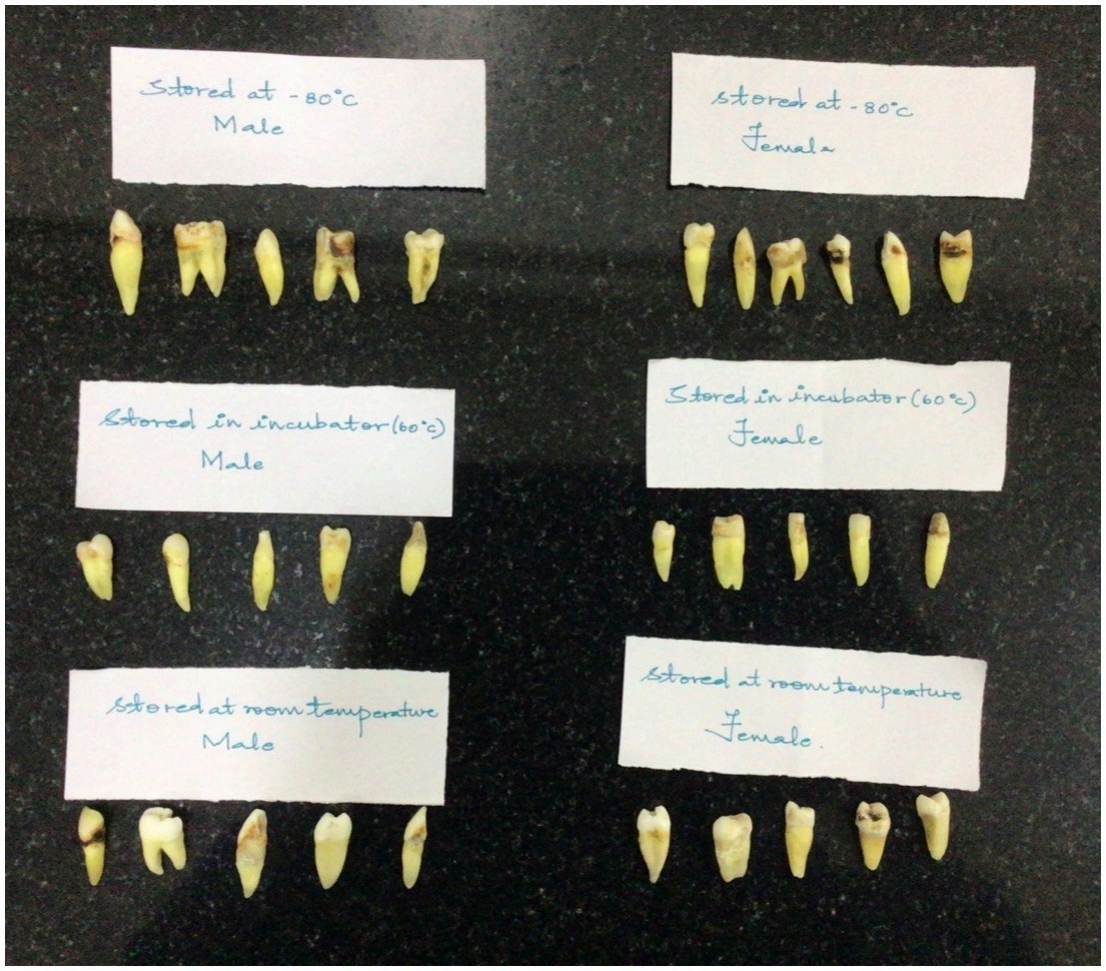
Teeth samples included in the study.

**Fig 2:**
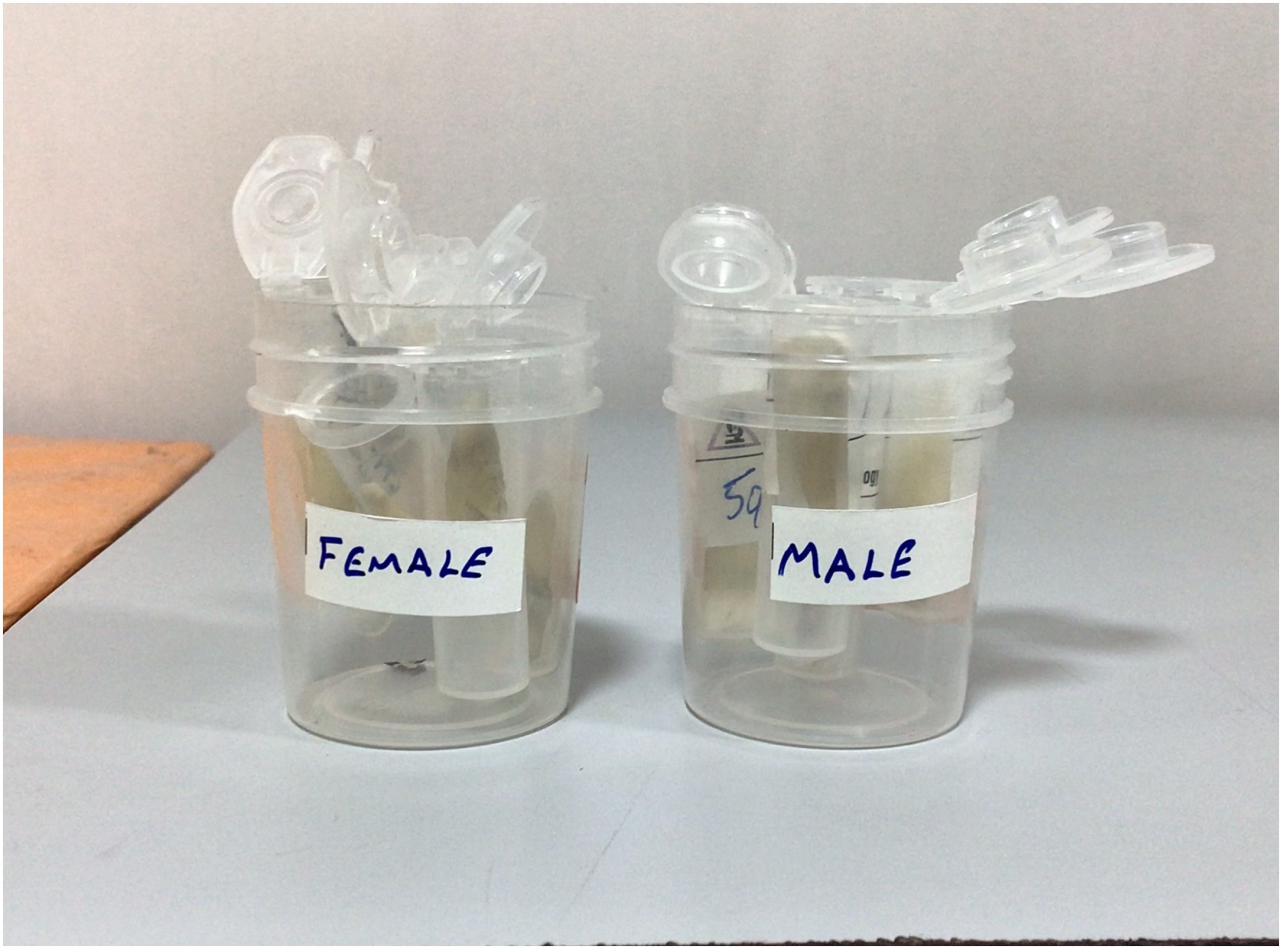
Stored at Room Temperature (Group 1).

**Fig 3:**
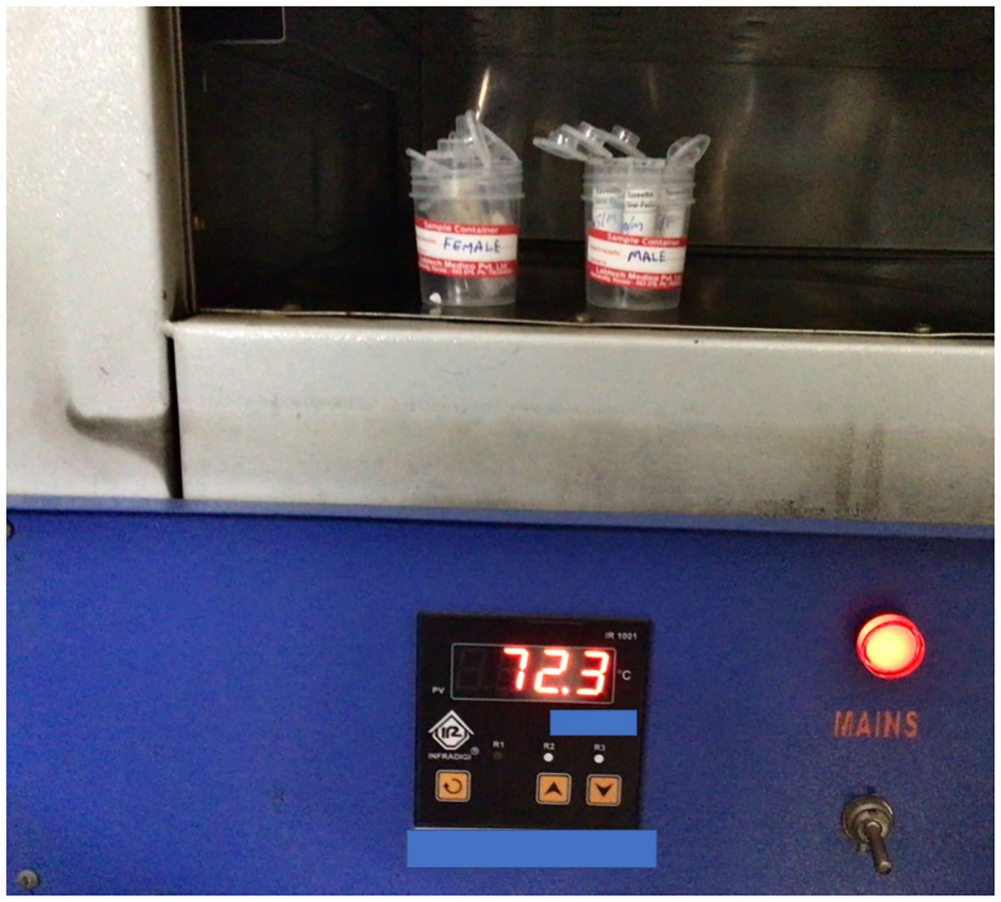
Stored at High Temperature (Group 2).

**Fig 4:**
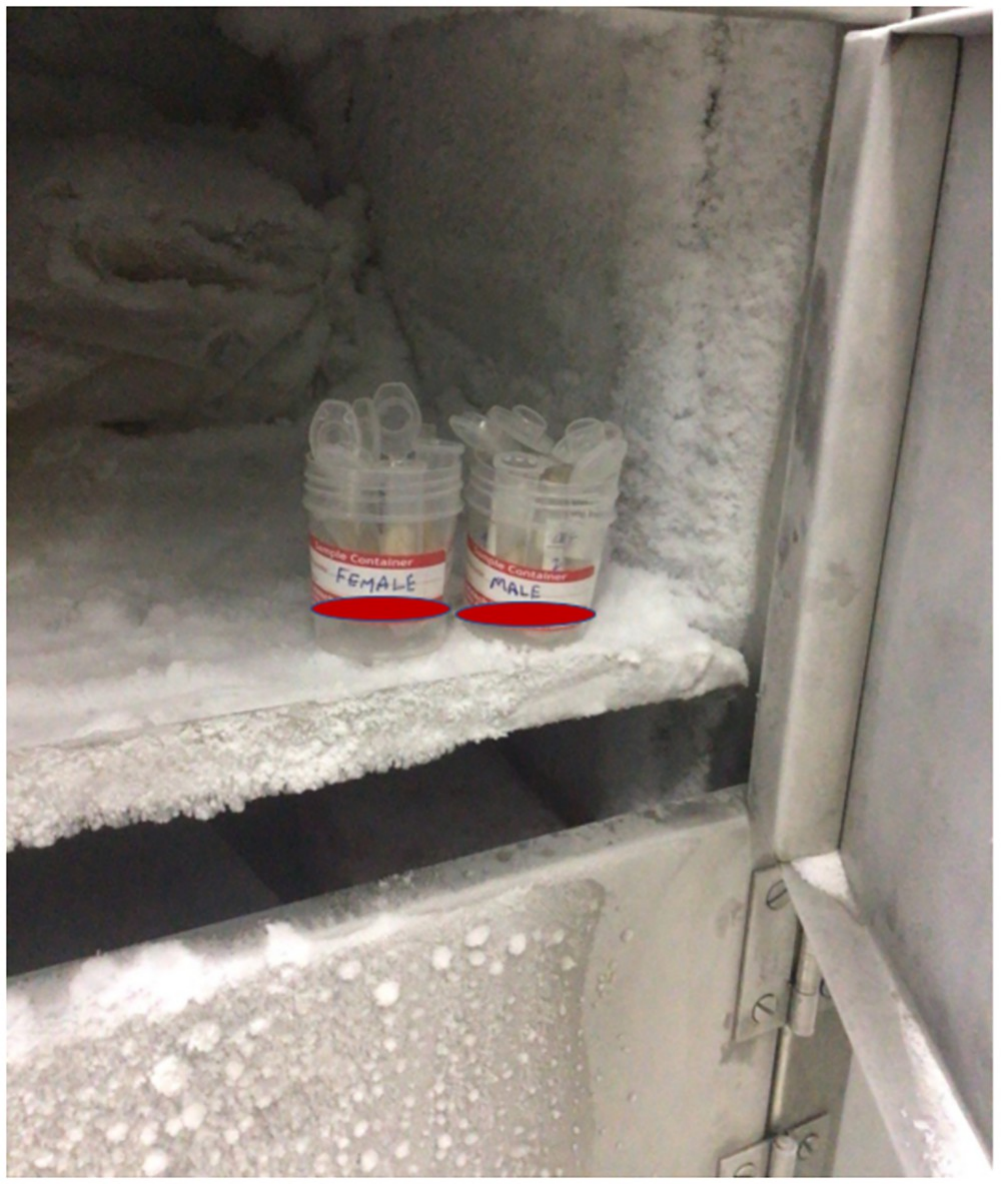
Stored at Frozen Temperature of -80 (Group 3).

Prior ethical clearance was obtained from Scientific Review Board—Saveetha Dental College and Hospitals, Chennai, India- IHEC/SDC/OPATH-2104/22/253 on 20/6/2022. After this, a pilot study was conducted in the Department of Oral Pathology, Saveetha Dental College and Hospital, Chennai, Tamilnadu, India. The study population was patients, both male and female, who underwent extraction for orthodontic and periodontic treatments. A random sampling technique was used to select the participants. A written informed consent was obtained from all individual participants included in the study. Additionally, patients signed informed consent regarding publishing their data and photographs. Each group included 10 samples, with 5 male and 5 female samples, resulting in a total of 30 teeth with vital pulps (Fig 1). Group 1 (R group) was maintained at room temperature of approximately 30°C (Fig 2), Group 2 (H group) at a high temperature of 70°C (Fig 3), and Group 3 (F group) at a freezing temperature of -80°C (Fig 4).

### Inclusion criteria

The freshly extracted permanent, non-carious, vital incisors, canines, premolars, and molars teeth for the orthodontic and periodontic treatment purpose of 18–40 years from both genders were included. Teeth that were non-vital, calcified, pulpal carious, attrited, fractures, discolored, cement or crown restored, Deciduous teeth, and those samples with unknown clinical data were excluded.

### Sample collection

**Fig 5:**
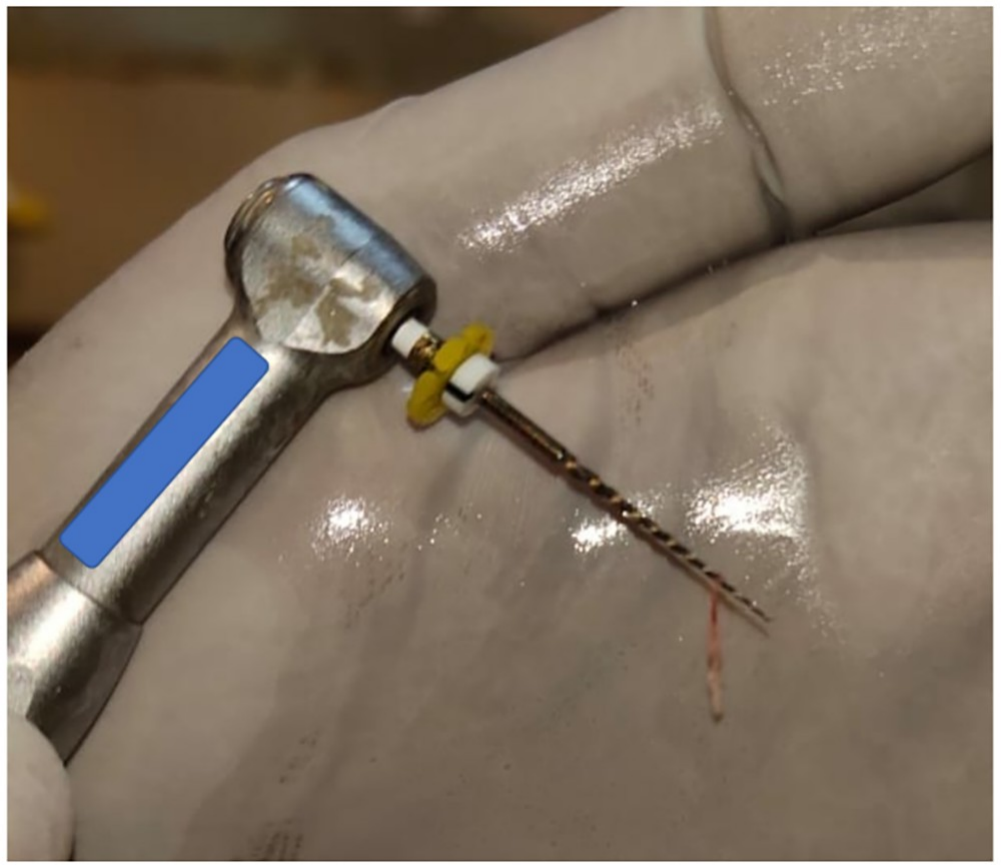
Extirpated pulp using endodontic instrument.

Once the selected tooth samples were aseptically extracted, they were decontaminated in a 1:10 solution of bleach for 30 min. No specific storage medium was used; instead, samples were stored in separate labelled. Eppendorf tubes to prevent cross-contamination. Subsequently, they were subjected to their respective allocated temperature for three weeks. Afterward, the pulp tissue was extirpated from all the tooth samples (Fig 5). The acquired tissue samples were kept in phosphate-buffered saline in individualized and labelled sterile Eppendorf tubes containing DNA extraction buffer.

### Sample analysis

#### Extirpation of dental pulp tissue

To extract the pulp tissue, endodontic access opening was performed using an endodontic spoon excavator and barbed broaches from the coronal pulp chamber and the root canal respectively (Fig 5). A minimum volume of 0.1mg of pulp was required for the molecular analysis. The collected samples were stored in phosphate-buffered saline in sterile Eppendorf tubes containing DNA extraction buffer and were labeled and transported to the lab for DNA analysis.

#### Isolation of DNA and amplification of SRY gene

DNA was isolated from the dental tissue using The PureLink^®^ Genomic kit. The genomic DNA was subjected to quantitative PCR for amplification of the sequence of interest using the SRY forward and reverse primers. No specific modifications were required in the PCR procedure for the samples that underwent various temperatures. Additionally, the Nanodrop One system from Thermo Scientific was also used for the quantity and quality of DNA while the concentration ranged from 62 ng/μL to 121 ng/μL, the DNA purity ranged from 1.69 to 1.78, Finally, the eluted DNA was stored at -20°C until further use. The eluted complementary DNA was run on an agarose gel electrophoresis to check the presence of SRY gene expression in the samples analyzed. A positive amplification with sequence- specific primers indicates the male sample and no amplification indicates the female sample. A known male DNA sample was used as the positive control, which showed amplification.

#### Statistical analysis

The data analysis was performed using SPSS version 26. The association between gene expression and gender was determined using the Kruskal-Wallis test. The concordance of the SRY gene expression with the gender was calculated and kappa statistics were done to assess the agreement between the gender and SRY gene expression.

## Results

In all the groups 7 out of 10 samples (70%) SRY gene amplification matched with gender recorded in the clinical data. Our findings were, Pulp tissue can be extracted from all teeth samples that were exposed to the room (approximately 30°C), 70°C, and -80°C. Interestingly the extirpation was easier and the amount of pulp tissue extracted from group 3 was greater than the other two. SRY genes could be amplified in all three groups. The false positives and False negatives of each group are given in Tables 1 and 2. Notably, all female samples were correctly identified in all study groups, with only one false result. SRY gene detected in the study samples identified accurate gender with a 46.70% Sensitivity and 93.30% Specificity (Tables 1 and 2). However, the chi-square test and Kruskal-Wallis test showed no significant difference in the correlation between gene expression and gender among the three groups (p = 1.000) (Tables 3 & 4).

**Table 1:** SRY gene expression analysis in % concordance with gender.

Groups	Gender		Male	Female
Room Temperature Gene Analysis	Male	Count	3	1
		% with Clinical Concordance	60.00%	20.00%
	Female	Count	2	4
		% with Clinical Concordance	40.00%	80.00%
High Temperature Gene Analysis	Male	Count	2	0
		% with Clinical Concordance	40.00%	0.00%
	Female	Count	3	5
		% with Clinical Concordance	60.00%	100.00%
Freezing Temperature Gene Analysis	Male	Count	2	0
		% with Clinical Concordance	40.00%	0.00%
	Female	Count	3	5
		% with Clinical Concordance	60.00%	100.00%
Gene Analysis	Male	Count	7	1
		% with Clinical Concordance	46.70%	6.70%
	Female	Count	8	14
		% with Clinical Concordance	53.30%	93.30%

Table 1 represents SRY gene expression analysis in % concordance with gender was assessed. For males % concordance of SRY gene with clinical data for Room Temperature, High Temperature and Freezing Temperature was 60% (n = 3), 40% (n = 2) and 40% (n = 3) and Overall concordance of SRY gene with male gender was 46.7%. For Females % concordance of SRY gene with clinical data for Room Temperature, High Temperature and Freezing Temperature was 80% (n = 4), 100% (n = 5) and 100% (n = 5) and Overall concordance of SRY gene with male gender was 93.30%.

**Table 2:** Sensitivity and specificity.

Diagnostic Test * Actual Gender Cross- Tabulation			Actual Gender		Total
			Negative	Positive	
Diagnostic Test	Negative	Count	14	1	15
		% within Diagnostic Test	93.30%	6.70%	100.00%
	Positive	Count	8	7	15
		% within Diagnostic Test	53.30%	46.70%	100.00%
Total		Count	22	8	30
		% within Diagnostic Test	73.30%	26.70%	100.00%

Table shows that the diagnostic test had higher specificity (True negatives) compared to sensitivity (True positives). There were more false positives than false negatives in the diagnostic test.

**Table 3:** No significant difference in correlation between the gene expression and gender between the three groups (p = 1.000).

Chi- Square	0.000
df	2
Asymp. Sig.	1.000

**Table 4:** Based on Kruskal-Wallis test no significant correlation between the gene expression and gene across categories of groups (p>0.005).

Null Hypothesis	Test	Significance	Decision
The distribution of correlation between Gene Expression and Gender is the same across categories of groups	Independent—Samples Kruskal-Wallis Test	1.000	Retain the null hypothesis

SRY gene expression analysis in % concordance with gender was assessed. For males % concordance of the SRY gene with clinical data for Room Temperature, High Temperature, and Freezing Temperature was 60% (n = 3), 40% (n = 2) and 40% (n = 3) and overall concordance of the SRY gene with male gender was 46.7%. For Females % concordance of the SRY gene with clinical data for Room Temperature, High Temperature, and Freezing Temperature was 80% (n = 4), 100% (n = 5), and 100% (n = 5) and Overall concordance of the SRY gene with female gender was 93.30% (Table 1). Kappa statistics value 0.4 for all three groups (Room Temperature, High Temperature, and Freezing Temperature) which is considered moderate to good agreement (Table 5).

**Table 5:** Kappa statistics value 0.4 for all three groups (Room Temperature, High Temperature and Freezing Temperature) which is considered moderate to good agreement.

Groups			Value	Asymptotic Standardized Error	Approximate Tb	Approximate Significance
Room Temperature	Measure of Agreement	Kappa	0.4	0.284	1.291	0.197
High Temperature	Measure of Agreement	Kappa	0.4	0.232	1.581	0.114
Freezing Temperature	Measure of Agreement	Kappa	0.4	0.232	1.581	0.114
Total	Measure of Agreement	Kappa	0.4	0.148	2.477	0.013

**Fig 6:**
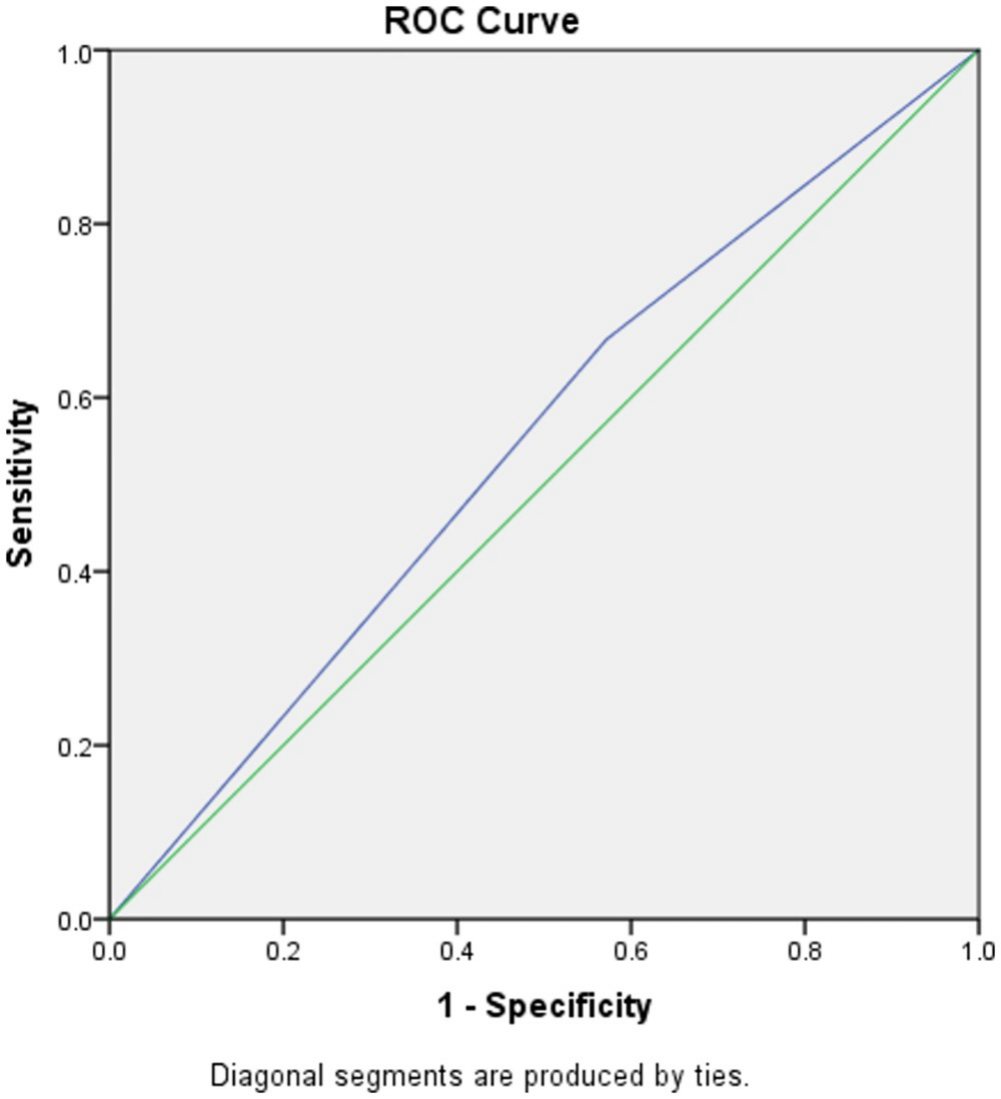
There is no significant area under the curve for the gene at Room Temperature in identifying the gender (p = 0.820). AUC is 54.8%.

**Fig 7:**
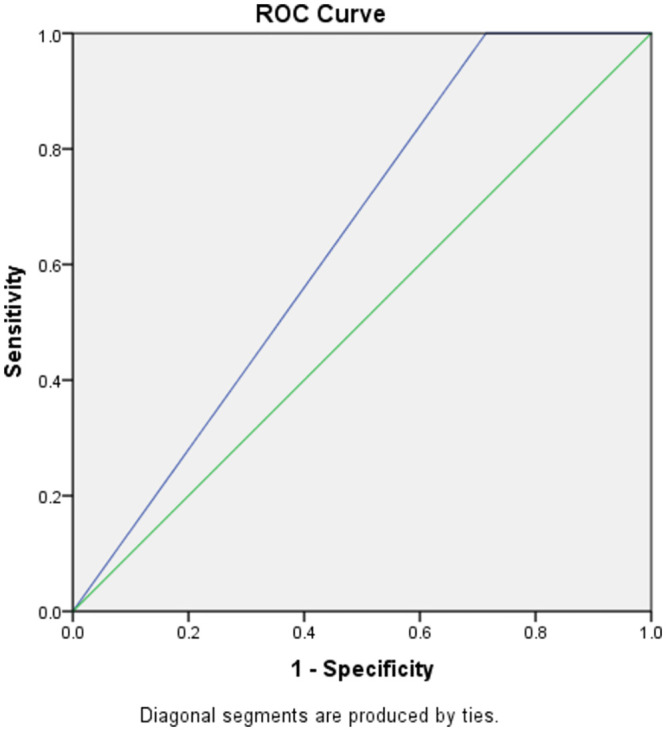
There is no significant area under the curve for the gene at High Temperature in identifying the gender (p = 0.494). AUC is 64.3%.

**Fig 8:**
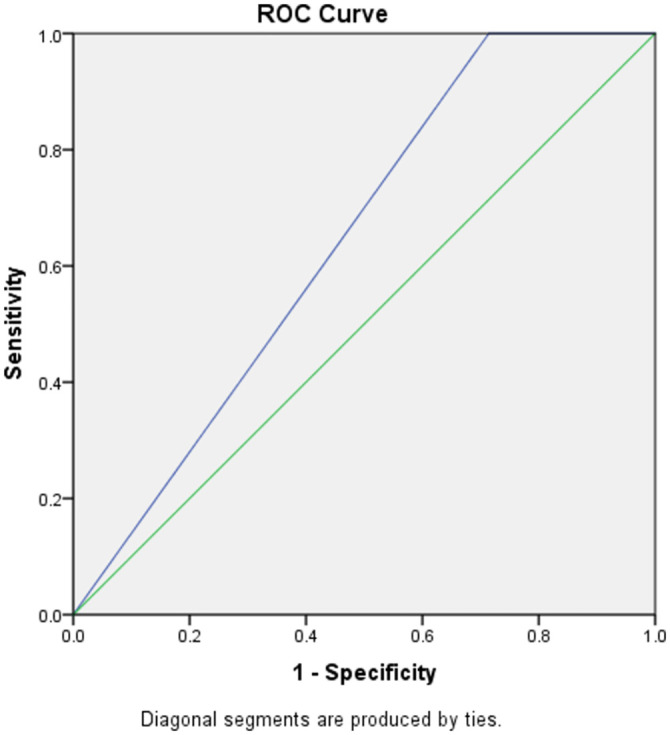
There is no significant area under the curve for the gene at Freezing Temperature in identifying the gender (p = 0.494). AUC is 64.3%.

Method AUC and Receiver operating characteristic (ROC) graphs indicate a valid model performance across the range of temperatures (Figs 6–8).

**Fig 9:**
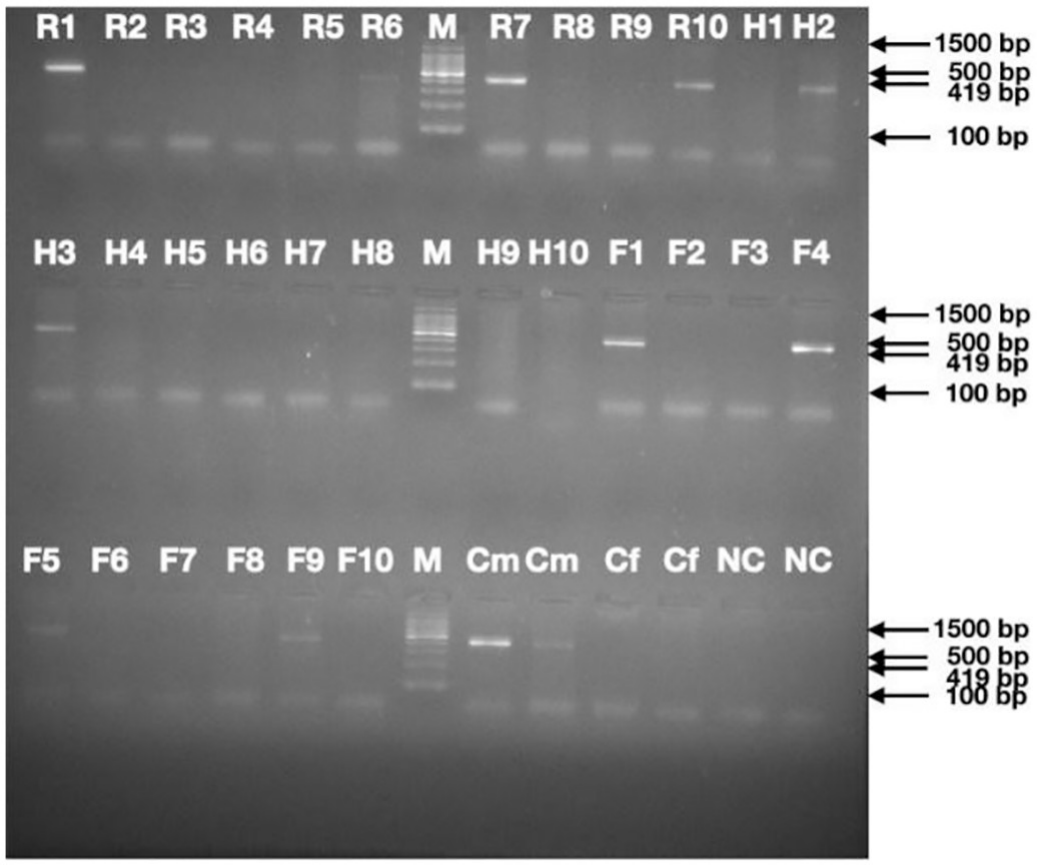
PCR for unknown dental pulp samples depicted in a gel electropherogram showing SRY amplicon of size 419bp.

Fig 9 is the electrophoretogram showing SRY amplicon of size 419 bp produced using sequence specific primers employing optimized reaction conditions (M—100 bp Standard DNA ladder). 7 out of 10 samples in Group I (Room Temperature) showed exact gender by SRY gene and 2 were False Positive (R5, R7) and 1 was False Negative (R9); 7 out of 10 samples in Group 2 (High Temperature) showed exact gender by SRY gene and 3 were False Negative (H7, H8, and H9); and 7 out of 10 samples in Group 3 (Freezing Temperature) showed exact gender by SRY gene and 3 were False Negative (F2, F3, and F8). The actual male gender samples were R1, R5, R6, R9, R10, H2, H3, H7, H8, H9, F1, F2, F3, F4 and F8. The actual female gender samples were R2, R3, R4, R7, R8, H1, H4, H5, H6, H10, F5, F6, F7, F9 and F10.

## Discussion

Gender determination is crucial for identifying a mutilated human body. Since teeth can withstand various temperatures without any effect on the encased pulp, the DNA extracted from the Pulp serves as a reliable source for genes including the Sex determining genes. Our study explored the SRY gene in the dental pulp DNA from adults who had their vital teeth extracted. Recent literature suggests that the SRY gene detected from the dental pulp can determine gender [8, 9]. Our study utilized PCR analysis on the DNA extracted from the dental pulp of vital permanent teeth without any anomalies and pathologies. Nevertheless, a few studies on teeth with anomaly and primary teeth were also shown to have 100% accuracy [11, 12]. However, Gopa and Hedge found a significant drop in accuracy to 40% with false positives when analyzing primary teeth after 6 months [11]. This implies that the choice of dentition for the gender prediction does not influence the gender determination using PCR- amplified genes.

In our study, the freshly extracted tooth stored in an empty Eppendorf under various temperatures for 3 weeks yielded sufficient Pulp. A minimum volume of 0.1mg of pulp was required for the PCR analysis and we successfully detected the SRY gene with moderate to good accuracy. Previous studies have established that storage methods like fresh water and soil [13], seawater [14], saline [15], natural soil [16] can yield DNA with 100% accuracy for gender determination. However, Sea water storage required modification of PCR cycles for effective DNA retrieval. buried teeth for 6 months had an accuracy drop to 92% and false PCR results were observed with freshwater samples compared to natural soil storage. Our study validates the possibility of storing the extracted teeth in an empty container for 3 weeks without any media as a choice of storage for gender determination using dental pulp.

Notably, in the present study, the storage period of 3 weeks did not influence the accuracy of the gene detected. However, Nayar 2017 found that the accuracy of AMEL gene detection for gender determination dropped to 73% when the number of storage days was increased from 2 days to 6 weeks in normal and saline water samples. This highlights that both the storage media /method and storage duration can influence the accuracy of gene detection in PCR [16].

Group 1 (kept at approximately 30°C) is at 70% accuracy on DNA retrieval. Pawar and More, [15] reported a significant drop of 94% accuracy for the samples kept at room temperature for 6 months. Group 2 and Group 3, where teeth were subjected to extreme temperatures also showed good to moderate accuracy in DNA retrieval and SRY gene detection.

Our study for the first time detected the SRY gene in the DNA amplified from a frozen pulp sample kept at -80 °C using PCR without demanding any procedural modification. With the method adopted, the SRY gene detected 7 out of 15 and 14 out of 15 female samples among all the 3 groups studied. The sensitivity and specificity obtained were 46% and 93.3%.

Previously, Gopa Kumar and Hegde also found false positives in their study conducted on the primary teeth [11]. However, our study did not find a statistically significant difference in gender prediction using the SRY gene across the three groups. Nevertheless, the results of the study showed moderate to good agreement with the gender recorded in the clinical data.

The current study is the first of its kind in storing the extracted tooth samples in an empty Eppendorf without any media with successful DNA retrieval across the study groups.

Additionally, our study for the first time analyzed the tooth kept under frozen temperature for the SRY gene detection. The limitations were the small sample size and not analyzing the concentration of the DNA yield. The future scope of research on gender determination studies on dental pulp could include large sample sizes, long storage durations at different temperatures, and pulp analysis for SRY gene expression.

## Conclusion

In conclusion, the current study observed that dental pulp tissue can be a reliable source for DNA extraction for forensic casework regardless of storage duration or environmental conditions, and that for gender identification, the SRY gene can be a reliable biomarker. Future research must include large sample sizes, different ages, hybrid environmental/ treatment, and different PCR methodologies.
