# Correction: SRY gene isolation from teeth for forensic gender identification—An observational study

**DOI:** 10.1371/journal.pone.0297835

**Published:** 2024-01-23

**Authors:** Prathibha Prasad, Mohamed Jaber, Dinesh Y., Prathibha Ramani, Abdulrahman Arafat, Abdalla Khairy

The Acknowledgments statement is missing in the published article. The Acknowledgments is as follows: The authors would like to express their appreciation to Ajman University for covering the Article Processing Charges (APC) associated with this research.
